# Supplementary material for: Patterns of Intron Gain and Loss in Fungi
Source: PLoS Biol. 2004 Nov 30;2(12):e422. doi: 10.1371/journal.pbio.0020422 (PMC532390; doi:10.1371/journal.pbio.0020422)
Supplement: Table S1 — Also available at http://genes.mit.edu/NielsenEtAl/. (4.3 MB ZIP). [file pbio.0020422.st001.zip › NielsenEtAl/html/395.html]

AN8559.1.NCU03913.1.MG06511.1.FG08622.1


```
 CLUSTAL W (1.82) Multiple Sequence Alignments - Introns Inserted


Sequence 1: NCU03913.1	417 aa
Sequence 2: FG08622.1	404 aa
Sequence 3: MG06511.1	429 aa
Sequence 4: AN8559.1	376 aa
Alignment Length: 440 aa
Number Identitical Residues: 175 aa
Alignment Score (without introns) 9181


MG06511.1 	-----MSLANASIDSV1TR~-----PLRGFLANSRPAVPSISKSQRQRLYSTHPPNARLN
NCU03913.1	MRGSTIRPKAQCLRSL~SK~GLTKRPSAGLRSVPVAATLAPSLPS-QRAYSTHPPHAKLN
FG08622.1 	-------MKHRVIHQF~KR~-------AHIASCPSTARFSLALPIGRRCYSTHPPNAKLN
AN8559.1  	-----MNFSAWGIKAV~QT1-------RLFAALPAFLCLGKQKGPRHLPFPQHHP-----
          	            :  .             : : .     .      :  :. * *     

MG06511.1 	VPIDYSTTPLLAHSSQTALSNPELSPEVRNGATKRMNLFQAINDALSTALAEDESVMLFG
NCU03913.1	LPTDYSTTPLLAQTSQSALNNPELSPEVRNGPTKRMNMFQAVNDALSVALAQDESVLIFG
FG08622.1 	LPVDYGTTPLLAHSSQTALSHKELPEHIRNGPTKKMNLFQAINDAMGIALTEDESVVVFG
AN8559.1  	-PFSYPRHG--THSTSLSSCRPLWPSKLLDGP-EPVNPPEPPN-RLQIDASPSPHPILPR
          	 * .*      ::::. :  .   . .: :*. : :*  :. *  :    : .   ::  

MG06511.1 	EDVAFGGVFRCSMGL~AEKHGGERVFNTPLCE~QGIMGFAIGMAAEGMRPVAEIQFADYV
NCU03913.1	EDVAFGGVFRCTGKL~ADTYGADRVFNTPLCE~QGIMGFAIGVAAEGMRPVAEIQFADYV
FG08622.1 	EDVAFGGVFRCTMNL~AETHGAERVFNTPLTE~QGIMGFGIGLAAEGMRPIAEIQFADYV
AN8559.1  	ELPEP------PTLH2NQRRTPHRPFQIGQ-E1QGIIGFAIGAAAEGMKPVAEIQFADYV
          	*          .     :    .* *:    * ***:**.** *****:*:*********

MG06511.1 	FPAFDQMVNEAAKFRYRDGANGRSAGGLTVRMPCGLVG~HGAL~YHSQSPESLFTHIPGF
NCU03913.1	YPAFDQLVNEAAKFRYRDGSCGRSAGGLTVRMPCGGVG~HGGL~YHTQSPESLFTHIPGL
FG08622.1 	YPAFDQLVNEAAKFRYRDGTCGRSVGGLTVRMPCGGVG~HGGL~YHSQSPESLFTHIPGL
AN8559.1  	FPAFDQIVNEAAKFRYREGATG------------GNAG1HGAL2YHSQSPEALFAHIPGL
          	:*****:**********:*: *            * .* **.* **:****:**:****:

MG06511.1 	RVVMPRSPVQAKGLLLAAIR-SNDPVVFMEPKVLYRAAVEQVPMASYTLPLSKAEILKEG
NCU03913.1	RVIMPRSPIQAKGLLLSAIR-SNDPCVFMEPKILYRAAVEQVPTGSYTLPLSKAEVLKEG
FG08622.1 	KVIMPRSPAQAKGLLLAAIR-SNDPCVFLEPKILYRAAVEQVPTGSYELPLSKAEVLKEG
AN8559.1  	QVVIPRSPSQAKGLLLASIFESKNPVVFMEPKVLYRAAVEHVPSEYYTIPLNKAEVIKPG
          	:*::**** *******::* .*::* **:***:*******:**   * :**.***::* *

MG06511.1 	KDLTIISYGQPLYICQNAIATAEKDLGISVELIDLRTIYPWDKECVFESVRKTGRAIVVH
NCU03913.1	KDVTIISYGQPLYKCMDALQKAEKDFGVSVELIDLRTVYPWDKETVFQSVRKTGRCIVVH
FG08622.1 	KDVTIVSYGQPLYLCHNAIKQAEQDLGISVELIDLRTLYPWDKKTVLESVRKTGRAMVVH
AN8559.1  	NDVTIISYGQPLYLCSAAIAAAEKNLGASVELIDLRTIYPWDRQTVLDSVNKTGRAIVVH
          	:*:**:******* *  *:  **:::* *********:****:: *::**.****.:***

MG06511.1 	ESMVNQGVGAEVAACIQEDADTFNRLEAPVERVAGWSIPTPLAFEKFNAPDAAS~KSFSS
NCU03913.1	ESMVNAGIGAEVAAAIQEDSETFVRLEAPVARVAGWSIHTPLMFEALNIPDVAR1-----
FG08622.1 	EAMVNAGIGAEVAAVIQEDHDTFLRLEAPVARVAGWSIHQPLLYERFNLPDVAR1-----
AN8559.1  	ESMVNFGVGAEVAATIQTG--AFLRLEAPVQRVAGWSTHTGLTYEKLILPDVTR1-----
          	*:*** *:****** ** .  :* ****** ******    * :* :  **.:       

MG06511.1 	RKFCHITRTDPLQTAGVYDRIKRVMEY
NCU03913.1	----------------IYANIKKVLNF
FG08622.1 	----------------IYDNIKRLLDY
AN8559.1  	----------------IYDAIKRTLEY
          	                :*  **: :::
```
